# The association between glutamine repeats in the androgen receptor gene and personality traits in dromedary camel (*Camelus dromedarius*)

**DOI:** 10.1371/journal.pone.0191119

**Published:** 2018-02-07

**Authors:** Sherif Ramadan, Amira M. Nowier, Yusuke Hori, Miho Inoue-Murayama

**Affiliations:** 1 Wildlife Research Center, Kyoto University, Kyoto, Japan; 2 Faculty of Veterinary Medicine, Benha University, Moshtohor, Egypt; 3 Biotechnology Research Department, Animal Production Research Institute, Dokki, Egypt; 4 Graduate School of Letters, Kyoto University, Kyoto, Japan; 5 Wildlife Genome Collaborative Research Group, National Institute for Environmental Studies, Tsukuba, Japan; Universite Clermont Auvergne, FRANCE

## Abstract

Temperament traits such as fearfulness are important as they define an animal’s responses to its environment and handling. The increasing automation of daily tasks and growing population limits contact between camels and humans. Such limitations contribute to fear of humans and changes in physical environment. Monoamine oxidase A (*MAOA*) and androgen receptor (*AR*) genes are important candidates associated with mammal personality. In our analysis, *MAOA* exon 15 showed no polymorphism but a novel polymorphism was seen in the camel *AR* exon 1; 16, 17, 18, and 19 glutamine repeats were detected. We genotyped 138 camels belonging to four Egyptian breeds: Maghrabi (*n* = 90), Sudani (*n* = 15), Somali (*n* = 23), and Baladi (*n* = 10) for *AR*. Out of the 90 genotyped Maghrabi camels, we evaluated responses of 33 and 32 mature females to a novel object and exposure to an unfamiliar person, respectively. *AR* gene showed a significant association based on the principal component (PC) score, which indicated the fear of human touch, and the PC score indicates fear during interaction with novel objects. Individuals carrying a shorter genotype in homozygote (*S/S*) were found to be more fearful. Furthermore, we found that Sudani and Somali breeds had a higher frequency of shorter genotype (*S/S*), which was associated with increased fearfulness. These findings reflect the behavioral tendency and consequently, affect the use of this breed. This is the first report showing the role of *AR* glutamine repeats influencing a behavioral trait in dromedary camels and leading to inter-breed differences. Fear-related traits reported here are important because camels cope with various types of stresses and fear, resulting from the demands of intensive production systems and racing events. However, further studies, employing functional genomics and linkage analysis are necessary for confirming the relationship between fearfulness and genetic variation.

## Introduction

Animal temperament influences individual behavioral changes in regard to stressors or environmental challenges, and impacts the adaptability of the animal to new environments, handling, animal welfare, and productivity [1]. Temperament traits such as fearfulness are important as they define the animal responses to the environment and handling [2]. Reduced animal fear and increased stress tolerance, for instance, towards humans and new management conditions, are crucial behavioral traits, without which animal domestication and breeding would be impossible [3].

Behavioral traits are defined by a combination of environmental and genetic factors. A genetic basis of animal behavior was indicated by breed differences [4], and by moderate to high heritability of temperament traits in various species [5]. Many studies have mapped the quantitative trait loci and single nucleotide polymorphisms for behavior-related traits [6–9]. The selection of desirable temperament traits is becoming increasingly important for farm animals [10]. On some farms, the milking temperament of dairy cattle is already included in the selection criteria of breeding programs [3], while in beef cattle, the ease of handling is being included in breeding programs [11].

Over the past few decades, new management systems for livestock production have been introduced worldwide. Camel management has been changing in recent years from an extensive to a semi-intensive or intensive system, and has presented new challenges for both the animal and farmer [12]. Increasing automation of daily processes, and growing camel herds limit the contact between camels and farmers, and contribute to an increased fear of humans and changes in physical environment [13, 14]. These behavioral challenges may adversely affect the life of the animal, and negatively impact their economically important production traits [1, 15, 16].

The Monoamine oxidase A gene (*MAOA*) codes for an enzyme that plays an important role in the breakdown of neurotransmitters, such as serotonin, dopamine, and norepinephrine, and maintains a constant level of these chemicals in the brain [17]. The MAOA gene in dromedary camels is located on the X chromosome, and is composed of 15 exons [18]. *MAOA* is one of the candidate genes associated with the personality of mammals, such as generalized anxiety and panic disorders, which are characterized by feelings of anxiety, fear, excessive worrying, and tension in the face of life experiences [19, 20]. Androgens play an important role not only in fertility, but also in modulating behavior in various species [21–23]. The human androgen receptor gene (*AR*) is located on the X chromosome, and has eight exons. It contains a highly polymorphic tri-nucleotide (CAG) repeat encoding glutamine in exon 1, and the length of this poly-glutamine repeat affects the level of transactivation [24]. Unlike the human *AR*, the dromedary camel *AR* is composed of nine exons [18]. Associations between glutamine repeat numbers of *AR* exon 1 and various behavioral traits have been documented in a number of animal species [25–27].

The dromedary camel (*Camelus dromedarius*) is a multipurpose animal. It provides milk, meat and wool, and can be used for agricultural work, transportation, racing, and beauty contests [28]. In Egypt, there are different dromedary camel breeds used for different purposes: Maghrabi for milk and meat production, Sudani and Somali for racing competitions, and Baladi (or Falahi) for agricultural work [29]. The different uses and the type of work can both affect the camel’s personality [30]; in humans, it was hypothesized that job characteristics and types of work are related to differences in personalities [31].

The objectives of the present study were as follows: first, to measure the fear response of the most important dromedary camel breed in Egypt (Maghrabi) to a novel object and an unfamiliar person; second, to estimate the polymorphism of the above-mentioned candidate genes among the four Egyptian camel breeds (Maghrabi, Sudani, Somali, and Baladi), and third, to investigate the possible association between genetic polymorphism and personality. To our knowledge, there is no existing study that has evaluated the genetic basis for the personality of dromedary camels.

## Materials and methods

### Subject animals

We used 138 dromedary camels (21 males and 117 females) belonging to four Egyptian breeds: Maghrabi (*n* = 90), Sudani (*n* = 15), Somali (*n* = 23), and Baladi (*n* = 10). The Maghrabi individuals were reared at the Camel Studies and Production Development Center in Matrouh Governorate, belonging to the Animal Production Research Institute (APRI), Agricultural Research Center, Egypt. All animals were identified by ear tags. Concentrate feed mixture (CFM) was offered each morning. The CFM was composed of 25% wheat bran, 25% yellow corn, 9% decorticated cotton seed meal, 20% barley, 15% rice bran, 3% molasses, 2% premix, and 1% common salt. Rice straw forage was offered to the animals in the afternoon. The Camel Studies and Production Development Center in Matrouh Governorate has a yard measuring about 50 × 60 m^2^ and 20 shaded rooms (6 × 6 m^2^) constructed with iron bars for housing six camels per room. The other three breeds (Sudani, Somali, and Baladi) were collected from five breeders in four governorates (Cairo, Giza, Behaira, and Sharkia) located in the Nile Delta in the northern part of Egypt.

Among the 90 Maghrabi individuals for which hair samples were collected, we assessed the fear response of 33 and 32 mature camels to a novel object (N.O.) and the approach of unfamiliar person (UF.P.) behavioral tests, respectively. The 32 individuals used in the UF.P. and 33 individuals in the N.O. partially overlapped. The assessed fear responses towards both, novel object and unfamiliar person, were video-recorded for three minutes from the start of the test. The age of tested individuals ranged from 6 to 13 years. All individuals were sexually matured and under similar management and feeding systems. They were not pregnant nor in their breeding season (November to March).

### Novel object test

The novel object (N.O.) test was conducted in order to investigate reactions related to fearfulness. The object used was a red pilates ball (60 cm diameter). The test was based on the novel object test for dairy cattle [32], which we modified for captive camels. The test arena was a section of the normal camel house, separated from other sections where herd mates were contained by iron bars to minimize disturbances during testing behavior in a new environment, and to minimize isolation stress. In this arena, the test animal could approach and investigate the novel object also. The camels were individually moved from the home pen to the test arena. In the arena, the camel was positioned facing the gate and the novel object was presented in front to ensure uniform visibility of the object. The 6 m × 6 m arena was divided into 16 squares (1.5 m × 1.5 m each). We measured eight behavioral items from the video recordings. The definitions and detailed information of measured behavioral attributes are shown in S1 Table.

### Approach by an unfamiliar person

In the same arena but on a different day, camels were evaluated for their reactions to an unfamiliar person. The test was based on studies on dairy cows and dogs [33, 34], which were modified to suit captive camels. The camels were individually moved from the home pen to the testing arena. In the arena, the camel was positioned in the center, with a gap of approximately two squares (3 m) between the unfamiliar person and the test camel. For the test, the unfamiliar person approached the camel and tried to touch the camel’s neck. We measured 10 behavioral attributes by observing video recordings. The definitions and detailed information of the measured behavioral attributes are shown in S2 Table.

### DNA extraction and genotyping

Genomic DNA from hair samples were extracted for the above-mentioned 138 camels using a QIAGEN DNeasy tissue kit (QIAGEN, Valencia, CA, USA). For genotyping of *MAOA* exon 15, the primer pair F: 5′-AAATCACCCACACCTTCTGG-3′ & R: 5′-CTGTTTCTTATGAGCACGTCAA-3′, and for *AR* exon 1, the following primer pair F: 5′-ACTTTCCCGGGCTTAAGCAG-3′ & R: 5′-GAAGCTGTTCCCCAGG-ACTC-3′, were designed using Primer 3 plus Version 2.3.6 software [35], based on the registered camel whole genome sequence (NW_011591769) [18]. We amplified a 406 bp fragment of exon 15 for *MAOA*, and a 316 bp fragment of camel *AR* exon 1. PCR was performed in a 15 μl reaction mix containing 20 ng of genomic DNA, 2x PCR buffer, all four dNTPs at 400 μM, each primer at 0.3 μM, and 0.5 U of LA*-Taq* DNA polymerase (TaKaRa, Shiga, Japan). The PCR cycle conditions for *MAOA* were as follows: initial incubation at 95°C for 2 min, followed by amplification for 35 cycles with 95°C for 30 s, 62°C for 45 s, 74°C for 60 s, and for *AR*, cycling at 95°C for 30 s, 61°C for 30 s, 74°C for 15 s, with a final extension at 74°C for 10 min was used. The amplified products were purified using a PCR purification kit (Roche, Mannheim, Germany), and the resultant products were sequenced using the same primers with Big Dye Terminator ver. 3.1 Cycle sequencing kit (Applied Biosystems, Foster City, CA, USA) according to the standard protocol, and electrophoresed on an ABI PRISM 3130xl sequencer (Applied Biosystems, Foster City, CA, USA). BLAST software [36] was used for sequence identification and confirmation. FinchTV 1.4.0 (Geospiza, Inc., Seattle, WA, USA; http://www.geospiza.com), MEGA 6 [37], and Bioedit 7.0.5.3 [38] software were used for sequence alignment and polymorphism detection. After confirming the polymorphism length due to different glutamine repeats, we designed a forward primer for *AR* labeled with 6-FAM, and genotyped the 138 samples on an ABI 3130xl DNA Sequencer (Applied Biosystems, Foster City, CA, USA), and the sizes of the fragments were estimated based on 400 HD ROX size marker using the GENEMAPPER software (Applied Biosystems, Foster City, CA, USA).

### Data analysis

We conducted principal component analysis (PCA) to contract the behavioral data obtained from the novel object and unfamiliar person tests, using the ‘prcomp’ function in the R software ver. 3. 1. 2 (R Foundation for statistical computing, Vienna, Austria). Correlation matrices were used for analysis. The number of components to be extracted was determined by parallel analysis. Principal component (PC) scores were calculated using the default steps of the ‘prcomp’ function. We genotyped the male and female camels, but because the small number of males, they were not included in the behavioral analysis. The association between genotypes and PC scores was tested by one-way analysis of variance (ANOVA) using the SAS software ver 9.1.3 (SAS Institute Inc, Cary, NC, USA)

### Ethical statements

All aspects of the study were performed according to the guidelines established by the Ministry of Education, Culture, Sports, Science and Technology in Japan (Notice No. 71). The protocol was approved by the Committee on the Ethics of Animal Experiments of the Wildlife Research Center, Kyoto University (Permit No. WRC-2017-005A). No special permission for behavioral research, such as this study, is requiredin Egypt.

## Results

### Behavioral traits

We constructed three principal components (PCs) for both the novel object and approach by unfamiliar person tests, which explained 89% and 83% of the total variance of the data for the two tests, respectively. The loading matrix of behavioral items on each PC is shown in Tables 1 and 2. The data from each PC was interpreted based on items that had high loading values (the absolute values that were higher than 0.50) on the PC.

**Table 1:** Loading score matrix of eight evaluation items of the novel object test (N.O.) for three principal components (PCs).

Item	*PC1*	*PC2*	*PC3*
Latency to approach N.O.	**0.68**	**0.51**	-0.34
Distance during interaction with N.O.	**0.85**	0.28	-0.25
Sniffing	**0.56**	**-0.77**	0.09
Moving head and neck	**0.90**	-0.11	-0.06
Moving whole body right and left	**0.89**	0.37	0.00
Moving whole body backward	**0.85**	-0.30	0.19
Latency to cross over the N.O.	**0.89**	-0.37	-0.07
Pattern to cross over N.O.	0.46	0.45	**0.76**
*Contribution ratio*	*0*.*60*	*0*.*19*	*0*.*10*
*Cumulative Contribution ratio*	*0*.*60*	*0*.*79*	*0*.*89*

Loading scores higher than 0.50 are in bold.

**Table 2:** Loading score matrix of 10 evaluation items of the approach of unfamiliar person test (UF.P) for three principal components (PCs).

Item	*PC1*	*PC2*	*PC3*
Camel accepted/not accept the touch of unfamiliar person	**-0.83**	0.34	0.16
Attempts to touch the camel	**0.93**	0.21	-0.08
Distance during interaction with UF.P	**0.90**	-0.18	0.08
Moving head and neck	**0.93**	0.21	-0.09
Moving whole body right and left	**0.93**	-0.16	0.17
Moving whole body backward	**0.86**	0.00	0.12
Duration of hand contact	**-0.92**	0.01	0.13
Urination and/or defecation	0.11	**0.60**	**0.55**
Teeth grinding	0.01	**0.61**	**0.73**
Vocalization	0.19	**0.78**	0.17
*Contribution ratio*	*0*.*57*	*0*.*16*	*0*.*10*
*Cumulative Contribution ratio*	*0*.*57*	*0*.*73*	*0*.*83*

Loading scores higher than 0.50 are in bold

In the novel object test, PC1 highlighted the fear of novelty because the seven behavioral items involved had high loading values. It indicates that individuals with high PC1 scores are more fearful when exposed to a novel object. PC2 explained the exploratory behavior, because it positively correlated with latency to approaching a novel object and negatively correlated with the number of sniffs. It indicates that individuals with high PC2 scores are less inquisitive. PC3 contained only one item depicting the fear response when crossing over a novel object. It indicates that individuals with high PC3 scores are more fearful when passing a novel object.

For the approach by unfamiliar person test, PC1 indicates the fear and avoidance of human touch, because it positively correlated with items involving human avoidance, and negatively correlated with items involving acceptance of human interactions. This indicates individuals with high PC1 scores are more fearful of humans. PC2 showed common signs of fear and anxiety, as it positively correlated with behavior related to fearfulness like urination, teeth grinding, and vocalization. Individuals having high PC2 scores are intimidated by approaching humans. PC3 indicated signs of fear similar to PC2, except for vocalization item that was not included in PC3.

### Genotyping

We sequenced a 406 bp fragment of exon 15 for *MAOA* (accession number: MF787280), but failed to detect any polymorphism in this region. However, we detected a novel length polymorphism in *AR*; at 316 bp, 319 bp, 322 bp and 325 bp of the alleles (accession numbers: MF542311, MF542314), respectively. For *AR*, the frequency of major (316 bp) and minor (322 bp) alleles were 0.45 and 0.015, respectively. We designated the major allele (316 bp) as short (*S*), and the other three alleles (319 bp, 322 bp and 325 bp) as long (*L*). Sequencing data suggested that the 316 bp, 319 bp, 321 bp, and 325 bp alleles contained 16, 17, 18, and 19 glutamine repeats, respectively. We divided the subjects into separate groups based on the possible interactions between *S* and *L* alleles; for instance, in the case of additive interaction, we designated them as *S/S*, *S/L*, and *L/L* groups. However, in the case of dominance of *L* over *S* or *S* over *L* alleles, we used two groups; with/without *L* where *S/L* and *L/L* were *L+*, *S/S* was *L-*, or with/without *S* where S*/S* and *S/L* were *S+*, *L/L* was *S-*, respectively. The *AR* genotype frequency among the four camel breeds was higher for the short genotype (*S/S*) in Sudani (0.692) and Somali (0.579) compared to the Maghrabi (0.192) and Baladi (0.286) breeds (Table 3). The allele frequencies of *AR* among the four camel breeds studied are shown in S3 Table. The genotypes and behavioral scores of all individuals are listed in S4 Table.

**Table 3:** Genotypes frequency of AR gene among the four Egyptian camel breeds.

Sex	Genotype	Maghrabi	Sudani	Somali	Baladi
Female		*n* = 78	*n* = 13	*n* = 19	*n* = 7
	*S/S*	0.192	0.692	0.579	0.286
	*S/L*	0.449	0.231	0.158	0.143
	*L/L*	0.359	0.077	0.263	0.571
Male		*n* = 12	*n* = 2	*n* = 4	*n* = 3
	*S/-*	0.417	0.500	0.250	0.333
	*L/-*	0.583	0.500	0.750	0.667

### Association analysis

**Fig 1:**
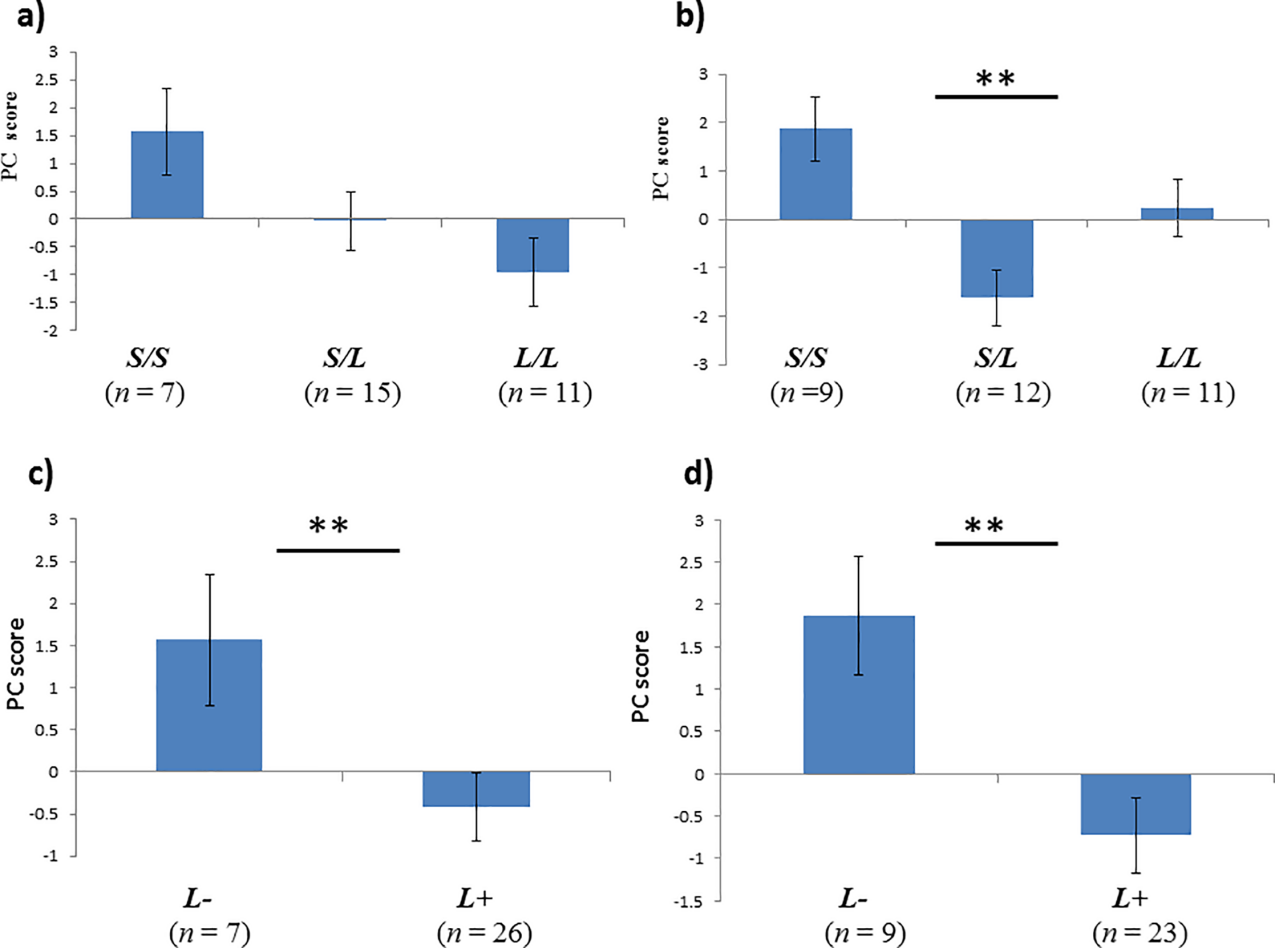
Scores of principal components (PCs). (a) PC1 of N.O. “additive interaction”, (b) PC1 of UF.P. “additive interaction”, (c) PC1 of N.O. “*L* dominant over *S*”, (d) PC1 of UF.P. “*L* dominant over *S*” for the AR genotypes. Error bars indicate standard error of the mean. Asterisks denote statistical significance: *P < 0.05, **P < 0.001.

We tested the association between the PC scores and genotypes of *AR* (Table 4). *AR* had significant association with PC1, which indicated a fear of human touch (*F* [2, 31] = 5.01, *P* = 0.002), and a marginal significant association with PC1, reflecting fear from and during interaction with the novel object (*F* [2, 32] = 3.21, *P* = 0.054) in case of additive interaction between *S* and *L* alleles. Highest score was observed for *S/S* genotype (Fig 1A and 1B, Table 4). The *AR* genotype showed a significant association with both PC1, indicating fear during exposure to novel object, as well as fear of human touch when *L* allele dominates over the *S* allele (*F* [1, 32] = 5.11, *P* = 0.031; and *F* [1, 31] = 9.79, *P* = 0.004, respectively). The S/S genotype displayed a higher score than the other genotypes (Fig 1C and 1D, Table 4). For the assumption involving *S* dominating over *L* allele, no significant associations with any of the PC scores were observed (Table 4).

**Table 4:** *P* values for the effect of AR genotypes on PCs of novel object and approach of unfamiliar person tests (additive and dominance interactions).

Allele interaction	Behavioral test	Number of genotypes	PC1	PC2	PC3
Additive	Novel object	(*S/S* = 7, *S/L* = 15, *L/L* = 11)	0.054	0.779	0.513
	Unfamiliar person	(*S/S* = 9, *S/L* = 12, *L/L* = 11)	0.002	0.620	0.834
*L* dominant	Novel object	(*L-* = 7, *L+* = 26)	0.031	0.909	0.649
	Unfamiliar person	(*L-* = 9, *L+* = 23)	0.004	0.324	0.879
*S* dominant	Novel object	(*S-* = 22, *S+* = 11)	0.078	0.484	0.410
	Unfamiliar person	(*S-* = 21, *S+* = 11)	0.710	0.666	0.552

## Discussion

We identified a significant association between fearfulness towards a novel object and unfamiliar person, as assessed from video recordings, to the *AR* glutamine repeat in Maghrabi camels. We observed a general trend in that individuals carrying the shorter genotype (*S/S*) reacted more to fear, from either the novel object or an unfamiliar person. In the case of fearfulness toward an unfamiliar person, *S/L* heterozygote individuals behaved differently and were less fearful than the *L/L* homozygote individuals. This result implies that additive interactions between *S* and *L* alleles are unclear, especially in the case of fearfulness in the unfamiliar person test. As a result, we assumed dominance of one allele (*L*) over another (*S*), and found a significant association with PC1 for both behavioral tests. In the case of dominance of *S* over *L* allele, there was no significant association detected with any of the PCs.

The association between androgens and various traits (infertility, fearfulness, and aggression) has been documented in a number of animal species [25, 39]. Humans and dogs with a shorter *AR* glutamine repeat tend to show more aggressiveness and impulsiveness [25, 40]. Previous reports have shown that the short glutamine repeat enhances the transcriptional activity of the androgen receptor by promoting interactions between the receptor and co-activators, meaning that the shorter *AR* allele exerts greater androgen activity [41]. Our results suggest that individual camels carrying shorter genotype (*S/S*) reacted intensely with fear upon exposure to a novel object and an unfamiliar person. As shorter *AR* alleles exert greater androgen activity, it might be expected that individuals carrying shorter genotypes show less fearfulness, and this trend does not seem to straightforwardly correspond to the previous patterns of associations in humans and dogs. Fearfulness is not the opposite of aggressiveness; fearfulness and aggressiveness are both negative reactions to novel objects and unfamiliar people. Although we did not measure the camels’ aggression in this study, fear response is able to be explained in accordance with aggression. On the other hand, some camels reacted positively to the previous two behavioral tests, and they scored low in their fear response.

The recent data on the associations between *AR* glutamine repeat polymorphism and *AR* function are somewhat contradictory. In vitro studies have shown that medium-length glutamine repeats had high transcriptional activity of *AR* compared with both shorter and longer glutamine repeats [42]. Sheppard et al. [43] found that the previous patterns of association in humans and dogs were not constant across different cell types. He found that the *AR* transcriptional activity was positively affected by an increased *AR* glutamine repeat length in skeletal muscle cells. Moreover, a positive association between the *AR* glutamine repeat and free testosterone in men has been observed in two large and independent population samples with more than 2,500 individuals [44]. Ito et al. [45] found a different pattern between allele length and aggression than that observed in humans and dogs. They found that locus *AR* glutamine repeats in three zebra species had longer alleles than those in horses. As zebras have not been domesticated it might be expected that they are more aggressive than horses and are expected to have shorter *AR* glutamine repeats. Finally, the low variation in camel *AR* glutamine repeats (16–19 repeats) compared to human (10–37 repeats) may cause minor variations in the *AR* activity, and there might be another locus in this gene which causes the major effect on the *AR* activity. Thus, the associations between *AR* glutamine repeat polymorphism and *AR* function are somewhat conflicting, and demand a more thorough hormonal investigation, such as measuring the circulating steroid of the tested individuals before conducting any behavioral tests. Moreover, these results should be tested in more camel populations with the help of functional genomics to confirm the trend of the association between *AR* activity and gene polymorphisms.

By genotyping other Egyptian camel breeds for *AR*, we found that both Sudani and Somali camels contained a higher frequency of shorter genotype (*S/S*), which was associated with higher fearfulness. The Maghrabi and Baladi camels contained lower frequency of shorter genotype (*S/S*). This indicates that Sudani and Somali camels respond strongly to fear either due to a novel object, or an unfamiliar person than the responses of Maghrabi and Baladi. This could be attributed to the fact that Baladi breeds are mainly used for agricultural work, whereas Maghrabi are bred for meat and milk production. During breeding, continuous contact between camels, humans, and inanimate objects exists. These conditions might accustom these two breeds to many activities and objects, and hence, less fearful. On the other hand, Sudani and Somali breeds were imported from Sudan and Somali African countries, and originally raised in large groups foraging natural pasture (pastoralism). Such isolation minimizes contact with different objects and people, thus causing anxiety and more fear upon exposure to strange events or environments.

## Conclusions

Our results suggest that a genetic polymorphism in *AR* glutamine repeat affects fear response towards novelty and unfamiliar person. Such polymorphism might be useful for a marker-assisted selection program for improvement of camel personality. We conclude that Sudani and Somali breeds show higher degrees of fear to novelty than the Maghrabi and Baladi breeds. This is the first evidence correlating polymorphism in androgen receptor gene and individual differences in behavioral traits in dromedary camels. Fear-related traits assessed in this study are important, because camels have to cope with various types of stress and fear, compounded by the demands of intensive production systems and racing events. However, further studies employing functional genomics and linkage analyses are necessary for validating the relationship between fearfulness and genetic polymorphism.

## Supporting information

S1 Table Observed behaviors in camels during exposure to the novel object (N.O.) test. (DOCX)

S2 Table Observed behaviors during approach and touch camel’s neck by unfamiliar person (UF.P.). (DOCX)

S3 Table Allele frequencies of AR gene among the four Egyptian camel breeds. *Numbers inside brackets = numbers of males.
(DOCX)

S4 Table Profiles of all samples. (DOCX)
