# Supplementary material for: The association between glutamine repeats in the androgen receptor gene and personality traits in dromedary camel (Camelus dromedarius)
Source: PLoS One. 2018 Feb 7;13(2):e0191119. doi: 10.1371/journal.pone.0191119 (PMC5802489; doi:10.1371/journal.pone.0191119)
Supplement: S1 Table — (DOCX) [file pone.0191119.s001.docx]

**S1 Table**

| **Parameters measured** | **Method** | **Description** |
| --- | --- | --- |
| 1. **Latency to approach N.O.** | Seconds | - Duration from the start of the test till the first trial to approach/touch the N.O. |
| 1. **Reaction to N.O. and it includes:-** | | |
| 1. Distance during interaction with N.O. | Near <1.5 m (code =1)  1.5 m< Moderate <3 m (code =2)  Far > 3 m (code =3) | - The distance between camel and N.O. during the interaction (sniffing, moving head and neck or moving the whole body). |
| 1. Sniffing | Frequency | - How many times the camel tried to explore the N.O by sniffing? |
| 1. Moving head (looking right/left) | Frequency | - How many times the camel looked and moved his head right and left during exploring N.O.? |
| 1. Moving whole body right and left | Frequency | - How many times the camel moved left and right during exploring N.O.? |
| 1. Moving whole body backward | Frequency | - How many times the camel moved backward during exploring N.O.? |
| 1. **Latency to cross over the N.O.** | Seconds | - Total time from the start of the test till crossing over the N.O.   (it includes latency to approach N.O. + time during interaction) |
| 1. **Pattern to cross over N.O.** | Slowly > 8 sec (code =1)  4 < Moderately < 8 (code =2)  Fast < 4 sec (code =3)  Jumping (code =4) | - The speed of the animal (Slow, Moderate, Fast) was calculated by dividing a fixed distance (10 m) over the measured time - The time was measured when the camel start crossing over the N.O. until it reached a defined marking (10 m distance after N.O.) |
