# Supplementary material for: The association between glutamine repeats in the androgen receptor gene and personality traits in dromedary camel (Camelus dromedarius)
Source: PLoS One. 2018 Feb 7;13(2):e0191119. doi: 10.1371/journal.pone.0191119 (PMC5802489; doi:10.1371/journal.pone.0191119)
Supplement: S2 Table — (DOCX) [file pone.0191119.s002.docx]

**S2 Table**

| **Parameters measured** | **Method** | **Description** |
| --- | --- | --- |
|  |  |  |
| 1. **Camel accepted the touch of unfamiliar person** | No (code = 1)  Yes (code = 2) | - The camel accepted the unfamiliar person touch to his neck or refused by moving left and right and backward |
| 1. **Attempts to touch the camel** | Frequency | - How many trails to succeed touching the camel’s neck by unfamiliar person - Maximum number of trails was 5 trials after that the animal was decided to be not touched |
| 1. **Distance during interaction with UF.P.** | Close < 1.5 m (code = 1)  1.5 m < Moderate < 3 m (code = 2)  Fare > 3 m (code = 3) | - The distance during the trails of unfamiliar person to touch camel’s neck - This indicates the camel’s fear, where the camel tries always to be away (by moving right, left, backward). |
| 1. **Response to the touching** | | |
| 1. Moving head | Frequency | - How many times the camel moved his head toward right/left direction during the trials of touch. |
| 1. Moving whole body right and left | Frequency | - How many times the camel moved left and right during the trials of touch. |
| 1. Moving whole body backward | Frequency | - How many times the camel moved backward during the trials of touch. |
| 1. **Duration of hand contact** | Not accept touching (code = 0)  Short <1 sec (code = 1)  Long > 1 sec (code = 2) | - Short: indicate that the unfamiliar person just succeeded to touch camel neck then the camel moved away or moved his neck away - Long**:** indicate that the unfamiliar person not only succeeded to touch camel neck but also catch the neck friendly for a period > 1 seconds |
| 1. **Signs of fearfulness** | | |
| 1. Urination and/or defecation | No (code = 1)  Yes (code = 2) | The camel urinating and/or defecating as a sign of fear |
| 1. Vocalization | No (code = 1)  Yes (code = 2) | Vocalization by the camel as a sign of fear |
| 1. Teeth grinding | No (code = 1)  Yes (code = 2) | Teeth grinding was observed in camels as a sign of fear and/or as a sign of aggressiveness and as a defensive mechanism |
