# Supplementary material for: The association between glutamine repeats in the androgen receptor gene and personality traits in dromedary camel (Camelus dromedarius)
Source: PLoS One. 2018 Feb 7;13(2):e0191119. doi: 10.1371/journal.pone.0191119 (PMC5802489; doi:10.1371/journal.pone.0191119)
Supplement: S3 Table — *Numbers inside brackets = numbers of males. (DOCX) [file pone.0191119.s003.docx]

**S3 Table**

| Breed/allele | Maghrabi | Sudani | Somali | Baladi |
| --- | --- | --- | --- | --- |
|  | *n* = 90 (12)^*^ | *n* = 15 (2) | *n* = 23 (4) | *n* = 10 (3) |
| 316 bp | 0.417 | 0.786 | 0.619 | 0.353 |
| 319 bp | 0.298 | 0.143 | 0.214 | 0.353 |
| 321 bp | 0.006 | 0.036 | 0.000 | 0.176 |
| 325 bp | 0.280 | 0.036 | 0.167 | 0.118 |
