# Supplementary material for: The association between glutamine repeats in the androgen receptor gene and personality traits in dromedary camel (Camelus dromedarius)
Source: PLoS One. 2018 Feb 7;13(2):e0191119. doi: 10.1371/journal.pone.0191119 (PMC5802489; doi:10.1371/journal.pone.0191119)
Supplement: S4 Table — (DOCX) [file pone.0191119.s004.docx]

**S4 Table**

| ID | Breed | Age/year | Sex | AR genotype | | Score of novel object test | | | Score of unfamiliar person test | | |
| --- | --- | --- | --- | --- | --- | --- | --- | --- | --- | --- | --- |
|  |  |  |  |  |  | PC1 | PC2 | PC3 | PC1 | PC2 | PC3 |
| 1 | Maghrabi | 13 | female | 316 bp | 319 bp | -1.385 | -0.123 | -0.604 | 0.331 | 3.270 | -1.285 |
| 2 | Maghrabi | 12 | female | 319 bp | 325 bp | -1.385 | -0.123 | -0.604 | -0.424 | 3.404 | -1.579 |
| 3 | Maghrabi | 13 | female | 316 bp | 325 bp | - | - | - | - | - | - |
| 4 | Maghrabi | 13 | female | 319 bp | 319 bp | - | - | - | - | - | - |
| 5 | Maghrabi | 12 | female | 316 bp | 316 bp | - | - | - | - | - | - |
| 6 | Maghrabi | 10 | female | 319 bp | 325 bp | - | - | - | - | - | - |
| 7 | Maghrabi | 13 | female | 316 bp | 325 bp | - | - | - | - | - | - |
| 8 | Maghrabi | 12 | female | 316 bp | 325 bp | - | - | - | - | - | - |
| 9 | Maghrabi | 11 | female | 316 bp | 316 bp | - | - | - | 1.762 | -0.284 | 0.110 |
| 10 | Maghrabi | 11 | female | 316 bp | 316 bp | - | - | - | - | - | - |
| 11 | Maghrabi | 11 | female | 316 bp | 319 bp | - | - | - | - | - | - |
| 12 | Maghrabi | 10 | female | 316 bp | 316 bp | - | - | - | - | - | - |
| 13 | Maghrabi | 10 | female | 316 bp | 316 bp | - | - | - | 3.477 | -0.644 | 0.693 |
| 14 | Maghrabi | 10 | female | 319 bp | 325 bp | -1.097 | -0.121 | -0.709 | 4.000 | 0.759 | 1.194 |
| 15 | Maghrabi | 11 | female | 316 bp | 325 bp | -0.113 | -1.407 | -0.703 | -2.849 | -0.144 | 0.795 |
| 16 | Maghrabi | 9 | female | 316 bp | 319 bp | -1.385 | -0.123 | -0.604 | -1.604 | 1.481 | 0.886 |
| 17 | Maghrabi | 9 | female | 316 bp | 319 bp | 0.671 | -0.594 | -0.036 | -2.940 | -1.114 | -0.343 |
| 18 | Maghrabi | 10 | female | 316 bp | 325 bp | -0.800 | 0.489 | 0.872 | 2.086 | -1.392 | -0.849 |
| 19 | Maghrabi | 8 | female | 325 bp | 325 bp | -0.369 | 0.769 | 0.861 | 2.929 | -0.578 | 0.489 |
| 20 | Maghrabi | 8 | female | 325 bp | 325 bp | -1.007 | 0.533 | 0.908 | 1.074 | 1.683 | 1.121 |
| 21 | Maghrabi | 9 | female | 316 bp | 325 bp | -0.734 | -1.047 | -0.534 | -2.940 | -1.114 | -0.343 |
| 22 | Maghrabi | 7 | female | 316 bp | 325 bp | -0.764 | -1.025 | -0.528 | -2.170 | 1.347 | -1.620 |
| 23 | Maghrabi | 7 | female | 316 bp | 325 bp | 2.630 | 0.848 | -1.340 | - | - | - |
| 24 | Maghrabi | 7 | female | 325 bp | 325 bp | -1.304 | -0.078 | -0.673 | -2.940 | -1.114 | -0.343 |
| 25 | Maghrabi | 8 | female | 319 bp | 325 bp | -1.196 | 0.205 | 0.152 | 2.610 | 0.011 | -0.348 |
| 26 | Maghrabi | 9 | female | 316 bp | 325 bp | 2.810 | 0.494 | 1.425 | - | - | - |
| 27 | Maghrabi | 7 | female | 316 bp | 325 bp | -1.060 | -0.913 | -0.475 | -1.572 | 1.494 | 0.885 |
| 28 | Maghrabi | 8 | female | 316 bp | 316 bp | 7.208 | 3.613 | -1.450 | 2.838 | -1.548 | -0.650 |

**S4 Table continued**

| ID | Breed | Age/year | Sex | AR genotype | | Score of novel object test | | | Score of unfamiliar person test | | |
| --- | --- | --- | --- | --- | --- | --- | --- | --- | --- | --- | --- |
|  |  |  |  |  |  | PC1 | PC2 | PC3 | PC1 | PC2 | PC3 |
| 29 | Maghrabi | 7 | female | 319 bp | 325 bp | -1.007 | 0.533 | 0.908 | -0.342 | -0.695 | -0.518 |
| 30 | Maghrabi | 8 | female | 316 bp | 316 bp | -0.681 | -0.322 | -0.784 | 2.432 | 0.021 | -3.035 |
| 31 | Maghrabi | 9 | female | 319 bp | 325 bp | -0.494 | -0.652 | 0.160 | -2.940 | -1.114 | -0.343 |
| 32 | Maghrabi | 7 | female | 316 bp | 319 bp | - | - | - | - | - | - |
| 33 | Maghrabi | 10 | female | 316 bp | 319 bp | -0.612 | -0.822 | -0.716 | -2.670 | -0.069 | 0.752 |
| 34 | Maghrabi | 11 | female | 316 bp | 316 bp | - | - | - | - | - | - |
| 35 | Maghrabi | 9 | female | 316 bp | 316 bp | 7.436 | -4.527 | 1.145 | 2.589 | -0.582 | 0.374 |
| 36 | Maghrabi | 9 | female | 319 bp | 319 bp | -1.034 | 0.295 | 0.015 | 2.796 | -0.651 | 0.464 |
| 37 | Maghrabi | 10 | female | 316 bp | 319 bp | -0.989 | 0.161 | 0.116 | -1.123 | -1.024 | -0.345 |
| 38 | Maghrabi | 8 | female | 316 bp | 316 bp | -1.007 | 0.533 | 0.908 | 2.291 | -1.482 | -0.854 |
| 39 | Maghrabi | 11 | female | 316 bp | 316 bp | -0.665 | -0.630 | 0.245 | 1.901 | 1.386 | 1.385 |
| 40 | Maghrabi | 12 | male | 325 bp | - | - | - | - | - | - | - |
| 41 | Maghrabi | 10 | female | 319 bp | 325 bp | -0.210 | 1.119 | 1.622 | -2.849 | -0.144 | 0.795 |
| 42 | Maghrabi | 7 | female | 316 bp | 325 bp | -0.180 | 1.097 | 1.617 | -0.982 | 0.107 | 0.593 |
| 43 | Maghrabi | 7 | female | 316 bp | 316 bp | -0.210 | 1.119 | 1.622 | 1.718 | -0.014 | 0.999 |
| 44 | Maghrabi | 8 | female | 316 bp | 319 bp | -1.304 | -0.078 | -0.673 | -2.940 | -1.114 | -0.343 |
| 45 | Maghrabi | 9 | female | 316 bp | 316 bp | -1.097 | -0.121 | -0.709 | -2.178 | -0.084 | 0.586 |
| 46 | Maghrabi | 8 | female | 319 bp | 321 bp | -1.385 | -0.123 | -0.604 | -1.373 | -0.057 | 0.679 |
| 47 | Maghrabi | 7 | female | 316 bp | 325 bp | 2.716 | 1.019 | -0.828 | - | - | - |
| 48 | Maghrabi | 3 | male | 319 bp | - | - | - | - | - | - | - |
| 49 | Maghrabi | 2 | female | 316 bp | 319 bp | - | - | - | - | - | - |
| 50 | Maghrabi | 4 | female | 316 bp | 325 bp | - | - | - | - | - | - |
| 51 | Maghrabi | 3 | female | 319 bp | 325 bp | - | - | - | - | - | - |
| 52 | Maghrabi | 2 | male | 325 bp | - | - | - | - | - | - | - |
| 53 | Maghrabi | 1 | female | 316 bp | 325 bp | - | - | - | - | - | - |
| 54 | Maghrabi | 4 | female | 316 bp | 319 bp | - | - | - | - | - | - |
| 55 | Maghrabi | 2 | male | 316 bp | - | - | - | - | - | - | - |
| 56 | Maghrabi | 3 | female | 316 bp | 325 bp | - | - | - | - | - | - |

**S4 Table continued**

| ID | Breed | Age/year | Sex | AR genotype | | Score of novel object test | | | Score of unfamiliar person test | | |
| --- | --- | --- | --- | --- | --- | --- | --- | --- | --- | --- | --- |
|  |  |  |  |  |  | PC1 | PC2 | PC3 | PC1 | PC2 | PC3 |
| 57 | Maghrabi | 4 | male | 319 bp | - | - | - | - | - | - | - |
| 58 | Maghrabi | 1 | female | 316 bp | 325 bp | - | - | - | - | - | - |
| 59 | Maghrabi | 2 | male | 319 bp | - | - | - | - | - | - | - |
| 60 | Maghrabi | 3 | female | 316 bp | 319 bp | - | - | - | - | - | - |
| 61 | Maghrabi | 4 | female | 319 bp | 325 bp | - | - | - | - | - | - |
| 62 | Maghrabi | 3 | male | 316 bp | - | - | - | - | - | - | - |
| 63 | Maghrabi | 4 | male | 316 bp | - | - | - | - | - | - | - |
| 64 | Maghrabi | 2 | female | 316 bp | 325 bp | - | - | - | - | - | - |
| 65 | Maghrabi | 1 | male | 316 bp | - | - | - | - | - | - | - |
| 66 | Maghrabi | 4 | male | 325 bp | - | - | - | - | - | - | - |
| 67 | Maghrabi | 2 | female | 316 bp | 316 bp | - | - | - | - | - | - |
| 68 | Maghrabi | 3 | female | 316 bp | 316 bp | - | - | - | - | - | - |
| 69 | Maghrabi | 4 | female | 319 bp | 319 bp | - | - | - | - | - | - |
| 70 | Maghrabi | 1 | female | 316 bp | 325 bp | - | - | - | - | - | - |
| 71 | Maghrabi | 4 | female | 316 bp | 319 bp | - | - | - | - | - | - |
| 72 | Maghrabi | 2 | female | 316 bp | 316 bp | - | - | - | - | - | - |
| 73 | Maghrabi | 3 | female | 325 bp | 325 bp | - | - | - | - | - | - |
| 74 | Maghrabi | 4 | female | 316 bp | 325 bp | - | - | - | - | - | - |
| 75 | Maghrabi | 2 | female | 316 bp | 319 bp | - | - | - | - | - | - |
| 76 | Maghrabi | 1 | female | 316 bp | 316 bp | - | - | - | - | - | - |
| 77 | Maghrabi | 1 | female | 316 bp | 316 bp | - | - | - | - | - | - |
| 78 | Maghrabi | 1 | male | 316 bp | - | - | - | - | - | - | - |
| 79 | Maghrabi | 2 | female | 319 bp | 319 bp | - | - | - | - | - | - |
| 80 | Maghrabi | 3 | female | 316 bp | 319 bp | - | - | - | - | - | - |
| 81 | Maghrabi | 4 | female | 319 bp | 319 bp | - | - | - | - | - | - |
| 82 | Maghrabi | 3 | female | 316 bp | 325 bp | - | - | - | - | - | - |
| 83 | Maghrabi | 2 | female | 316 bp | 316 bp | - | - | - | - | - | - |
| 84 | Maghrabi | 2 | female | 316 bp | 316 bp | - | - | - | - | - | - |

**S4 Table continued**

| ID | Breed | Age/year | Sex | AR genotype | | Score of novel object test | | | Score of unfamiliar person test | | |
| --- | --- | --- | --- | --- | --- | --- | --- | --- | --- | --- | --- |
|  |  |  |  |  |  | PC1 | PC2 | PC3 | PC1 | PC2 | PC3 |
| 85 | Maghrabi | 1 | male | 316 bp | - | - | - | - | - | - | - |
| 86 | Maghrabi | 4 | female | 319 bp | 319 bp | - | - | - | - | - | - |
| 87 | Maghrabi | 2 | female | 316 bp | 325 bp | - | - | - | - | - | - |
| 88 | Maghrabi | 2 | female | *319 bp* | 325 bp | - | - | - | - | - | - |
| 89 | Maghrabi | 1 | female | 316 bp | 319 bp | - | - | - | - | - | - |
| 90 | Maghrabi | 1 | female | 319 bp | 319 bp | - | - | - | - | - | - |
| 91 | Sudani | 9 | female | 316 bp | 316 bp | - | - | - | - | - | - |
| 92 | Sudani | 12 | male | 319 bp | - | - | - | - | - | - | - |
| 93 | Sudani | 12 | female | 316 bp | 316 bp | - | - | - | - | - | - |
| 94 | Sudani | 13 | female | 316 bp | 319 bp | - | - | - | - | - | - |
| 95 | Sudani | 12 | female | 316 bp | 316 bp | - | - | - | - | - | - |
| 96 | Sudani | 7 | female | 316 bp | 316 bp | - | - | - | - | - | - |
| 97 | Sudani | 12 | male | 316 bp | - | - | - | - | - | - | - |
| 98 | Sudani | 9 | female | 316 bp | 316 bp | - | - | - | - | - | - |
| 99 | Sudani | 12 | female | 316 bp | 321 bp | - | - | - | - | - | - |
| 100 | Sudani | 11 | female | 316 bp | 316 bp | - | - | - | - | - | - |
| 101 | Sudani | 10 | female | 316 bp | 319 bp | - | - | - | - | - | - |
| 102 | Sudani | 7 | female | 316 bp | 316 bp | - | - | - | - | - | - |
| 103 | Sudani | 7 | female | 319 bp | 325 bp | - | - | - | - | - | - |
| 104 | Sudani | 9 | female | 316 bp | 316 bp | - | - | - | - | - | - |
| 105 | Sudani | 7 | female | 316 bp | 316 bp | - | - | - | - | - | - |
| 106 | Somali | 10 | female | 316 bp | 316 bp | - | - | - | - | - | - |
| 107 | Somali | 11 | female | 319 bp | 325 bp | - | - | - | - | - | - |
| 108 | Somali | 12 | female | 316 bp | 316 bp | - | - | - | - | - | - |
| 109 | Somali | 9 | female | 316 bp | 316 bp | - | - | - | - | - | - |
| 110 | Somali | 8 | female | 316 bp | 316 bp | - | - | - | - | - | - |
| 111 | Somali | 7 | female | 316 bp | 316 bp | - | - | - | - | - | - |
| 112 | Somali | 5 | female | 316 bp | 316 bp | - | - | - | - | - | - |

**S4 Table continued**

| ID | Breed | Age/year | Sex | AR genotype | | Score of novel object test | | | Score of unfamiliar person test | | |
| --- | --- | --- | --- | --- | --- | --- | --- | --- | --- | --- | --- |
|  |  |  |  |  |  | PC1 | PC2 | PC3 | PC1 | PC2 | PC3 |
| 113 | Somali | 10 | female | 316 bp | 319 bp | - | - | - | - | - | - |
| 114 | Somali | 9 | Male | 325 bp | - | - | - | - | - | - | - |
| 115 | Somali | 10 | Male | 325 bp | - | - | - | - | - | - | - |
| 116 | Somali | 8 | female | 316 bp | 316 bp | - | - | - | - | - | - |
| 117 | Somali | 7 | female | 319 bp | 325 bp | - | - | - | - | - | - |
| 118 | Somali | 7 | female | 316 bp | 319 bp | - | - | - | - | - | - |
| 119 | Somali | 7 | female | 316 bp | 319 bp | - | - | - | - | - | - |
| 120 | Somali | 8 | female | 316 bp | 316 bp | - | - | - | - | - | - |
| 121 | Somali | 10 | female | 316 bp | 316 bp | - | - | - | - | - | - |
| 122 | Somali | 11 | female | 319 bp | 319 bp | - | - | - | - | - | - |
| 123 | Somali | 12 | female | 325 bp | 325 bp | - | - | - | - | - | - |
| 124 | Somali | 13 | Male | 319 bp | - | - | - | - | - | - | - |
| 125 | Somali | 7 | female | 316 bp | 316 bp | - | - | - | - | - | - |
| 126 | Somali | 8 | female | 316 bp | 316 bp | - | - | - | - | - | - |
| 127 | Somali | 10 | Male | 316 bp | - | - | - | - | - | - | - |
| 128 | Somali | 11 | female | 319 bp | 325 bp | - | - | - | - | - | - |
| 129 | Baladi | 9 | Male | 319 bp | - | - | - | - | - | - | - |
| 130 | Baladi | 4 | female | 319 bp | 319 bp | - | - | - | - | - | - |
| 131 | Baladi | 10 | Male | 321 bp | - | - | - | - | - | - | - |
| 132 | Baladi | 8 | female | 319 bp | 319 bp | - | - | - | - | - | - |
| 133 | Baladi | 7 | female | 316 bp | 316 bp | - | - | - | - | - | - |
| 134 | Baladi | 12 | female | 316 bp | 316 bp | - | - | - | - | - | - |
| 135 | Baladi | 5 | Male | 316 bp | - | - | - | - | - | - | - |
| 136 | Baladi | 7 | female | 321 bp | 321 bp | - | - | - | - | - | - |
| 137 | Baladi | 7 | female | 319 bp | 325 bp | - | - | - | - | - | - |
| 138 | Baladi | 8 | female | 316 bp | 325 bp | - | - | - | - | - | - |
